# Plague risk in vulnerable community: assessment of *Xenopsylla cheopis* susceptibility to insecticides in Malagasy prisons

**DOI:** 10.1186/s40249-017-0356-5

**Published:** 2017-11-07

**Authors:** Adélaïde Miarinjara, Jean Vergain, Jean Marcel Kavaruganda, Minoarisoa Rajerison, Sébastien Boyer

**Affiliations:** 1 Unité d’Entomologie Médicale, Institut Pasteur de Madagascar, Ambatofotsikely, 101 Antananarivo, PO box 1274 Madagascar; 2 Ecole Doctorale Sciences de la Vie et de l’Environnement, Université d’Antananarivo, Antananarivo, Madagascar; 3 Délégation Régionale pour l’Océan Indien, Comité International de la Croix-Rouge (CICR), 112, Rue Rainandriamampandry, Lot II B 16 – Faravohitra, 101 Antananarivo, Madagascar; 4 Unité Peste, Institut Pasteur de Madagascar, Ambatofotsikely, 101 Antananarivo, PO box 1274 Madagascar

**Keywords:** Plague, Prison, Flea, Insecticide, Madagascar

## Abstract

**Background:**

Prisons in Madagascar are at high risk of plague outbreak. Occurrence of plague epidemic in prisons can cause significant episode of urban plague through the movement of potentially infected humans, rodents and fleas. Rodent and flea controls are essential in plague prevention, by reducing human contact with plague reservoirs and vectors. Insecticide treatment is the key step available for the control of rat fleas which transmit the disease from infected rodents to human. The implementation of an adapted flea control strategy should rely on the insecticide susceptibility status of the targeted population. For the purpose of plague prevention campaign in prisons, we conducted insecticide resistance survey on *Xenopsylla cheopis,* the rat flea.

**Methods:**

Fleas were collected on rats caught in six prisons of Madagascar. They were exposed to insecticide treated filter papers and mortality was recorded following World Health Organization protocol.

**Results:**

The fleas collected in the prisons had different resistance patterns, while a high level of resistance to insecticides tested was described in the Antanimora prison, located in the heart of Antananarivo, the capital of Madagascar.

**Conclusions:**

This finding is alarming in the context of public health, knowing that the effectiveness of flea control could be jeopardized by insecticide resistance. In order to establish more accurate rat fleas control in prisons, the main recommendations are based on continuous monitoring insecticide susceptibility of flea, insecticide rotation, and the development of a new method for flea control.

**Electronic supplementary material:**

The online version of this article (10.1186/s40249-017-0356-5) contains supplementary material, which is available to authorized users.

## Multilingual abstracts

Please see Additional file 1 for translations of the abstract into six official working languages of the United Nations.

## Background

Plague is a highly transmissible disease caused by *Yersinia pestis*, a zoonotic bacterium that usually infects small mammals and their fleas [1]. Humans are extremely susceptible to plague and can get the disease by infected flea bites. This is the most common way of transmission between humans and infected rodents. When bubonic plague develops into the pneumonic form, inter-human airborne transmission may take place, and causing an epidemic of primary pneumonic plague among close contact [2]. The risk of transmission is important when epizootic plague kills susceptible rodent population. Hence infected fleas are in search of a new host such as humans. Thus, vector control must be prioritized to control the transmission. Rodent control may be the second step; since killing rodents without adequate flea control can increase transmission to humans, by spreading potentially infected fleas [2, 3].

Over the past four years, Madagascar has been the country most affected by plague [4]. The latter is endemic in Madagascar, above 800 m of altitude [5]. Mahajanga, a port city located in the western part of Madagascar has also been reported as a plague focus. In rural plague foci, the black rat, *Rattus rattus* is admitted as the main reservoir of plague, and associated flea species are known as the plague vectors [5]. *Xenopsylla cheopis,* the rat flea is accepted as the main vector, mainly found inside the human habitations, harbored by *R. rattus.* In urban areas, *R. norvegicus* replaces *R. rattus.* The former is more resistant to plague infection. This fact can explain very few reports of human cases and no epizootic is observable, despite a high seroprevalence in these rodents [6].

Plague is a disease of poverty in Madagascar, chiefly which threatens people from poor rural settlements. Yet in urban areas, severe overpopulation, lack of sanitation and hygiene in slum areas are chief factors related to plague outbreak [4]. Bubonic is the most common form of plague encountered in Madagascar, highlighting the promiscuity between humans, rodents and fleas.

These interactions are exacerbated in detention centers in Madagascar. According to a communication from the Malagasy Prison authorities, the maximum capacity of detention centers was about 10,360, but it actually houses 20,605 detainees [7]. In all prisons in Madagascar, hygiene conditions are very poor and sanitation facilities are insufficient [8]. According to Rubini et al. in 2016, the mortality risk factors associated with plague in the European medieval city can be found today in Malagasy detention centers [9]. In addition, rats are pests of everyday life in prisons [10]. Then in a case of plague outbreak, infected fleas can spread from the prison environment to the surroundings, with rats themselves, prisoners, prison guards, and visitors [9].

Human ectoparasites such as louse, lice and bed bugs are recurrent problems in prisons [11]. Insecticide treatments have been conducted to reduce the inconvenience caused by parasites. In such environment, insecticide selection pressure could be very high among insects, due to the frequent use of insecticide. Then generalized development of resistance as a result of selection for certain genes could occur amongst insect population [12]. Insect resistance to insecticides could explain the failure of insect control in prisons. Nevertheless, insecticide treatment is the only weapon available against rat fleas. In Madagascar *X. cheopis* in many plague foci was found to be resistant to 12 different insecticides, with different levels of resistance depending on the study sites [13]. In a context of public health emergency and with regard to the high epidemic risk of plague encountered in detention centers in Madagascar, a campaign was launched to control rodents and fleas in many detention centers, mainly in Antanimora prison, located in the center of the capital [10]. Many prisons in the country have benefited from this preventive campaign. However, there is no data on the sensitivity of rat fleas in prisons. We will present here the results of insecticide bioassays conducted on fleas collected from six prisons of Madagascar, during the flea and rodents control conducted in 2012.

## Methods

### Study sites

**Fig. 1:**
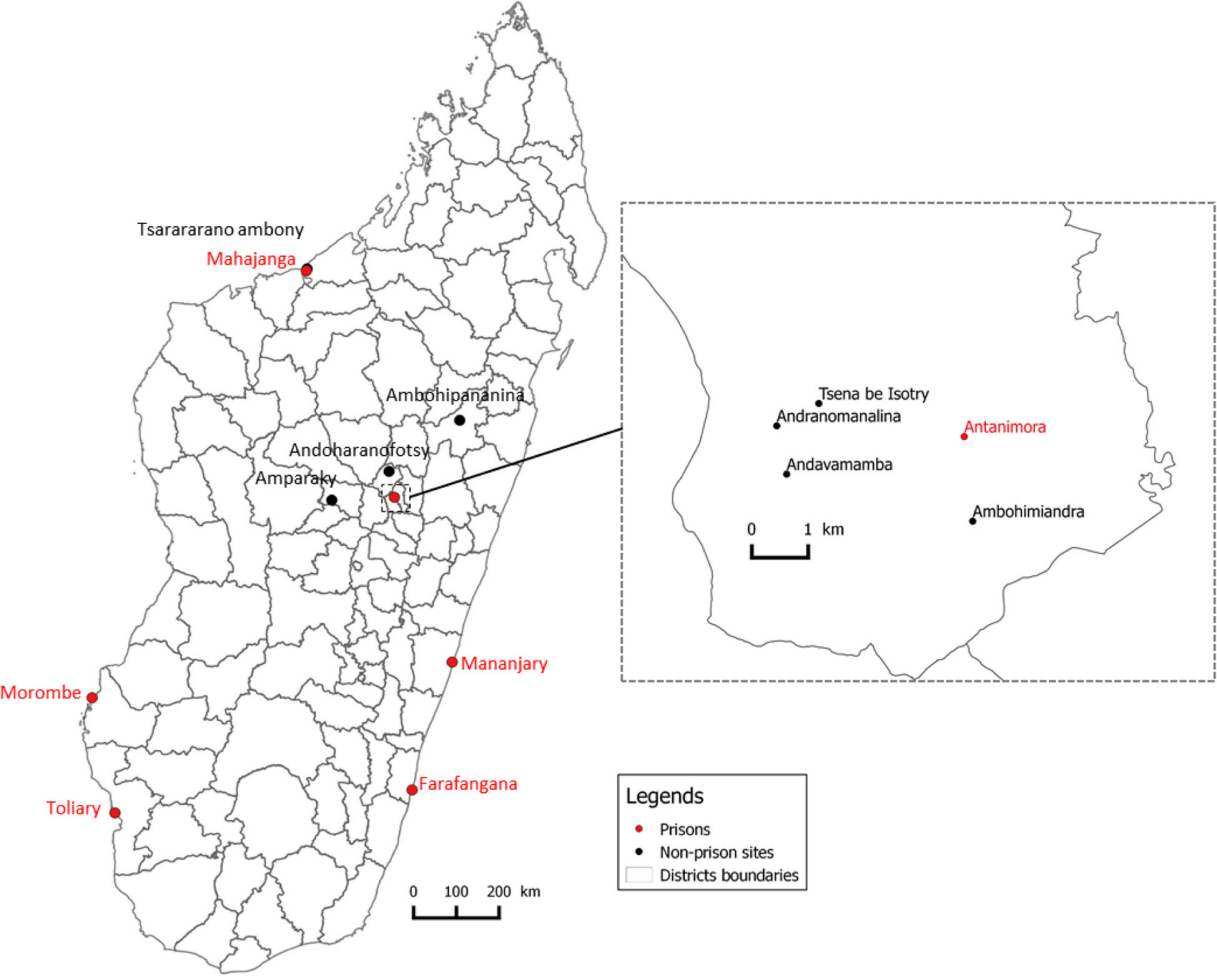
Map representing study sites mentioned in the study. In red: prisons where fleas were collected. In black: non-prison study sites where fleas were collected

Six detention centers were investigated between May and November 2012 (Fig. 1). Two prisons are located in plague endemic area: Antanimora prison in the capital Antananarivo, and Mahajanga prison in the harbor city of the same name, located on the western part of Madagascar. The prisons of Farafangana, Toliary, Manajary and Morombe are located outside plague endemic areas. No case of plague has been declared from the detention centers of Madagascar.

### Rodent trapping and flea collection

Rodents were trapped alive with wire mesh BTS traps (Besancon Technical Service, Besancon, France) settled in the evening and left overnight. Traps were baited with onions and dried fish during three consecutive nights. Fleas were combed from their rodents host and transported to the laboratory in a jar containing rice bran, covered with fine mesh tissue. Fleas were morphologically identified to species and reared in insectarium [14] until the required numbers of individuals for insecticide bioassays were obtained.

### Insecticide bioassays

Insecticide bioassays were conducted according to World Health Organization (WHO) protocol [15]. Laboratory bioassays conditions were described in Miarinjara et al., 2016 [13]. Briefly, fleas were exposed to strips of insecticide impregnated paper during diagnostic time. For each insecticide test, impregnated paper provided by WHO were used (Vector Control Research Unit, Penang, Malaysia). Four replicates of ten fleas per tube were exposed to impregnated papers and negative controls consisted in two replicates exposed to papers treated with insecticide solvent only. We tested insecticide belonging to the families used in insect pest control, namely pyrethroids (0.05% deltamethrin, 0.05% lambdacyhalothrin, 0.15% cyfluthrin, 0.75% permethrin, 0.025% alphacypermethrin and 0.5% etofenprox), carbamates (0.1% bendiocarb and 0.1% propoxur), organochlorine (4% DDT and 4% dieldrin) and organophosphates (5% malathion and 1% fenitrothion). Numbers of dead and paralyzed fleas were counted during exposure time. Final mortality was recorded after 24 h and compared with ANOVA tests. In addition, for each insecticide tested mean mortality of fleas from prisons was compared (ANOVA test) with that recorded in some localities from previous studies (black points on Fig. 1) [13]. The analysis was performed with R software (R Version 3.1.1 2014) and RStudio environment (R Studio Version 0.98.976 2009–2013).

## Results

**Fig. 2:**
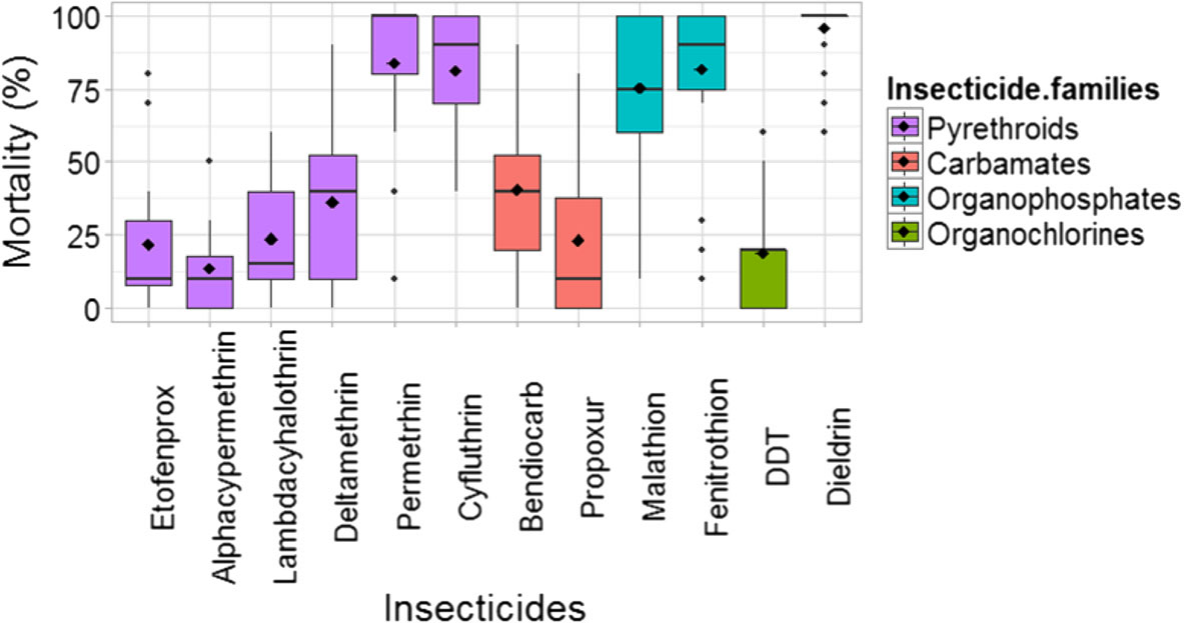
Box-and-whisker plot of mortality rate after 24 h for each insecticide for all prison populations. Diamond-shaped points inside the boxes are mean values. Horizontal bars in boxes are the 50th percentiles (medians), and the bottom and the top of the box represent the 25th and the 75th percentiles, respectively. The two limits of vertical lines above and at the bottom of the box are the whiskers and represent the maximum and the minimum values of the data. Points outside the limit of vertical line are “outlier”, which are values outside 95% the confidence interval

**Fig. 3:**
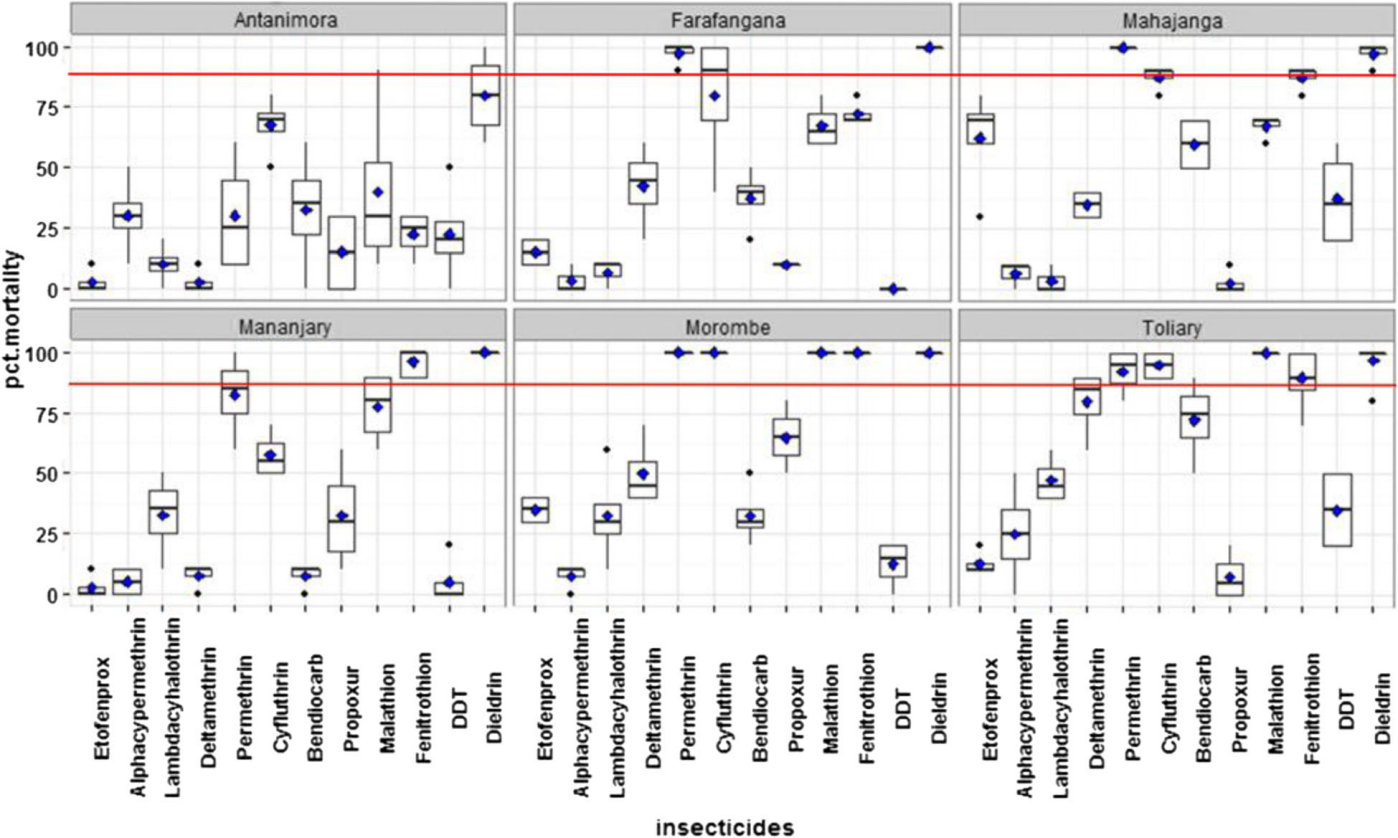
Details of mortality rate after 24 h, for each prison and insecticide tested. Red line represents the 80% mortality threshold

Fleas from detention centers investigated were resistant to the large majority of tested insecticides (alphacypermethrin, lambdacyhalothrin, etofenprox, deltamethrin, DDT, propoxur and bendiocarb). Highest mortality rates were obtained with dieldrin (95.9% ± 10.1), permethrin (83.7% ± 27.8), fenitrothion (81.6% ± 25.4), cyfluthrin (81.25% ± 19.4) and malathion (75.4% ± 25.7) (Fig. 2). Different profiles of susceptibility were observed according to prison and tested insecticides (Fig. 3). Notwithstanding the fleas from Antanimora, the main prison, all tested flea populations were susceptible to dieldrin, which is still the most efficient, even banned from many countries worldwide. Permethrin was still efficient in 4/6 prisons, while some populations resistant to permethrin were still susceptible to fenitrothion. Lowest mortality rates were observed in Antanimora prison, where fleas were resistant to all tested insecticides. Fleas from Morombe prison were still susceptible to at least four insecticides.

**Fig. 4:**
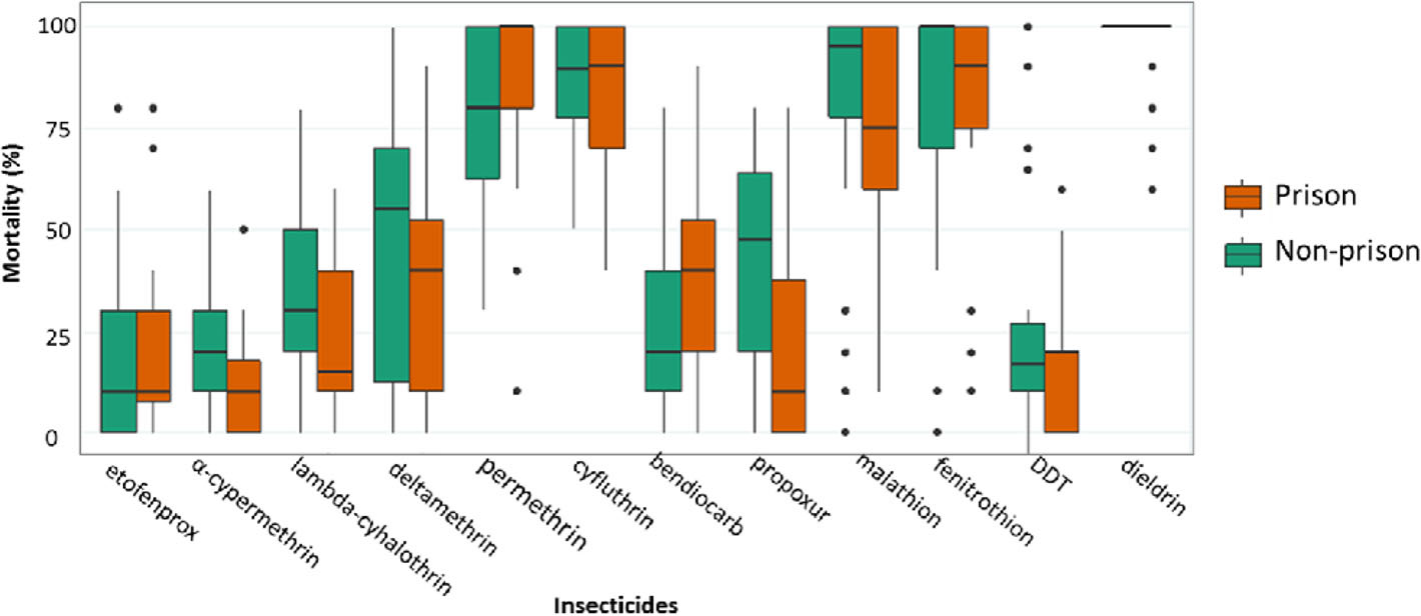
Comparison of mortality rate after 24 h for each insecticide for prison and non-prison flea populations

By comparing flea mortalities recorded in prisons and those recorded in plague surveillance areas (non-prison) where plague occurred or have been suspected, no significant difference in mortalities exists between prison and non-prison flea population (Fig. 4). The average mortality profile was the same for all tested insecticides.

## Discussions

This study is the first assessing the susceptibility of rat fleas in prisons. In this study, *X. cheopis* populations from Malagasy prisons were resistant to at least seven insecticides out of twelve. However resistance profile was very different from a prison to another. Few insecticides were still working (dieldrin, permetrin, cyfluthrin and fenitrothion) depending on prison. These insecticides may be recommended for the future intervention against fleas, vector of *Y. pestis*. Still, fleas from Antanimora prison, located in the capital showed high rate of resistance to insecticides, when compared with the other prisons. This population may have been subjected to higher insecticide pressure [12]. As this detention center is located in the capital, it can be obvious that insecticide products are more available. On the other hand, fleas from prisons located far away from the capital (Morombe and Toliary), showed less resistance to at least four insecticides (dieldrin, permethrin, cyfluthrin and fenitrothion).

Compared to previous results obtained with eight populations tested [13], the same profile of resistance was observed in fleas from prisons. However, it was assumed that insecticide selection pressure may be higher in prison due to frequent insecticide treatment against ectoparasites [16, 17]. Still, these results suggested that insecticide treatment in prisons is not as frequent as supposed and might not induce higher selection pressure on rat fleas. In large and overcrowded places of detention, external actors (International and Non-Gouvernemental Organizations) can as well support by organizing regular or punctual vector control campaigns.

As public health recommendations, policy of rat’s flea control and insecticide resistance management must be held in place. This policy may take in account, first, the results from insecticide bioassays. Each decision on the use of insecticide may be linked to data on the susceptibility of the targeted population. Hence it is important to make continuous surveillance of insecticide susceptibility of fleas from prisons, and gather many data as possible in other prisons.

Second, it is admitted that frequent use of the same insecticide leads to the establishment of resistant population [16, 17]. Experience from malaria vectors showed that the rotational use of insecticide of different modes of action could be done in order to keep their efficiency [12]. In this study, besides the case of Antanimora prison, alternating the use of pyrethroid (permetrin) and organophosphate (fenitrothion) can be recommended.

Third, WHO recommended the use of insecticide dust belonging to three chemical types (carbamate, organophosphate and pyrethroid) to fight against rat’ fleas [18]. For highly insecticide resistant population, such as fleas from Antanimora, other approaches on flea control must be taken into account. Systemic insecticide might be a promising alternative to target on host fleas [19–21]. Systemic insecticides are toxic for fleas when ingested by rodents, which make them more accurate on targeting rodents’ fleas than insecticide dusting. Fipronil, a phenylpyrazole insecticide, is considered as a good candidate in controlling rodent’s flea [21]. Besides, its chemical structure is different from insecticide recommended for flea control in plague foci, then can be efficient in localities where cross resistance is suspected among vectors. Besides, its systemic action is significant at low concentration [19]. Efficiency of systemic insecticides was tested on laboratory and feasibility on field was evaluated [19, 22–26]. This method relies on the palatability and the attractiveness of the bait toward the targeted rodent. So as to reduce rodents and fleas populations at the same time, systemic insecticide can be combined with slow-acting rodenticide [20].

Finally, as for improvement of resistance surveillance, knowing the insecticide resistance mechanism can accurately help finding out the most adapted insecticide for each scenario. However, hitherto very few details are known about insecticide resistance mechanisms involved for *X. cheopis*.

## Conclusions

Resistance to insecticides could be a serious challenge in detention centers. Finding an effective insecticide product is crucial in an emergency context to deal with a potential epidemic occurring in prisons. Our results suggest that insecticide treatment of rat fleas differ from one prison to another. The study of the mechanisms involved in resistance of fleas to insecticide can be carried out and alternative vector control policies should be considered. In addition, prisoner intoxication is a concern and must be taken into account in the choice of product and method to be adopted. However, improving the living conditions of inmates is a key factor in reducing contact with rodents and their fleas.

## Additional file

Additional file 1: Multilingual abstracts in the six official working languages of the United Nations. (PDF 622 kb)
