# Supplementary material for: Plague risk in vulnerable community: assessment of Xenopsylla cheopis susceptibility to insecticides in Malagasy prisons
Source: Infect Dis Poverty. 2017 Nov 7;6:141. doi: 10.1186/s40249-017-0356-5 (PMC5674827; doi:10.1186/s40249-017-0356-5)
Supplement: Additional file 1: — Multilingual abstracts in the six official working languages of the United Nations. (PDF 622 kb) [file 40249_2017_356_MOESM1_ESM.pdf]

Translation of the abstract into the six official working languages of the United Nations

خطر الطاعون في المجتمعات الضعيفة: تقييم حساسيةبرغوث الجرذ الشرقي للمبيدات الحشرية في السجون المدغشقرية

أديلاييد ميارينجارا ، جان فيرجين، جان مارسيل كافاروغندا، مينواريسوا راجيريسون، بوير سيباستيان

ملخص
خلفية: <bx/>السجون في مدغشقر معرضة بشكل كبير لتفشي الوباء. يمكن أن يتسبب تواجد وباء الطاعون في السجون في حدوث انتشار كبير للطاعون الحضري من خلال حركة الأشخاص والقوارض والبراغيث المحتمل إصابتهم بالعدوى تعتبر مكافحة القوارض والبراغيث أساسية في الوقاية من الوباء وذلك بتقليل اتصال الإنسان مع حاملي وخازني المرض. العلاج بالمبيدات الحشرية هي الخطوة الرئيسية المتاحة لمكافحة الفئران والبراغيث التي تنقل المرض من القوارض المصابة للإنسان. يجب أن يعتمد تنفيذ استراتيجية مكيفة لمكافحة البراغيث على مدى حساسية الفئة المستهدفة للمبيدات الحشرية. من أجل حملة الوقاية من الوباء في السجون، أجرينا استقصاء حول مقاومة المبيدات الحشرية على برغوث الجرذ الشرقي، برغوث الفئران.
الأساليب: <bx/> جمعت البراغيث من فئران تم إمساكها من ستة سجون في مدغشقر. تم تعريضها لمرشحات معالجة بالمبيدات الحشرية وتم تسجيل معدل الوفيات حسب بروتوكول منظمة الصحة العالمية.
النتائج: البراغيث التي تم جمعها في السجون كان لديها أنماط مقاومة مختلفة، تم تسجيل نسبة مقاومة عالية للمبيدات الحشرية في سجن أنتانيمورا، الواقع في قلب مدينة أنتاناناريفو عاصمة مدغشقر.
الاستنتاجات: هذه النتيجة مثيرة للقلق في سياق الصحة العامة، لأن فعالية مكافحة البراغيث يمكن أن تتعرض للخطر بسبب مقاومة المبيدات الحشرية. من أجل إنشاء نظام مكافحة للجرذان أكثر دقة في السجون، تشير التوصيات الرئيسية للرصد المستمر لحساسية البراغيث للمبيدات الحشرية وتنويع المبيدات الحشرية وتطوير طريقة جديدة لمكافحة البراغيث.

Translated from English version into Arabic by afaf benhadi, through

TRANSLATORS
WITHOUT BORDERS

弱势群体感染鼠疫风险：评估马达加斯加监狱印鼠客蚤（*Xenopsylla cheopis*）对杀虫剂的敏感性

Adélaïde Miarinjara, Jean Vergain, Jean Marcel Kavaruganda, Minoarisoa Rajerison, Sébastien Boyer

摘要
引言：马达加斯加监狱暴发瘟疫的风险很高。监狱流行的鼠疫可通过潜在感染的人类、啮齿动物和蚤类的流动引起重大的城市鼠疫疫情。控制啮齿动物和蚤类从而减少人类与鼠疫宿主和病媒的接触在鼠疫预防中是必不可少的。杀虫剂处理是控制鼠蚤的关键步骤，因为它能够防止疾病从受感染的啮齿动物传播到人类。恰当的鼠蚤控制策略的实施应以鼠蚤群体对杀虫剂敏感性状况为依据。为了在监狱中开展预防鼠疫的活动，我们对鼠蚤（*Xenopsylla cheopis*）进行了杀虫剂抗性调查。
方法：在马达加斯加 6 所监狱收集鼠蚤。根据世界卫生组织的标准操作流程，采用药膜法测定蚤类抗药性，并记录死亡率。

结果：监狱里收集的鼠蚤表现出不同的抗性模式。马达加斯加首都塔那那利佛中心地带的Antanimora监狱中的鼠蚤对杀虫剂有高水平抗性。
结论：这一发现为公共卫生敲响了警钟，因为蚤类对杀虫剂的抗性可能会削弱对蚤类防制的有效性。为了在监狱中建立更加精确的鼠蚤控制策略，建议持续监测蚤类的杀虫剂抗性、更替使用杀虫剂，以及开发新的鼠蚤控制方法。

Translated from English version into Chinese by Xin-Yu Feng, edited by Pin Yang

CDC CHINESE CENTER FOR DISEASE CONTROL AND PREVENTION
NATIONAL INSTITUTE OF PARASITIC DISEASES

Le risque de peste dans les communautés vulnérables: évaluation de la vulnérabilité aux insecticides de la *Xenopsylla cheopis* dans les prisons malgaches.

Adélaïde Miarinjara, Jean Vergain, Jean Marcel Kavaruganda, Minoarisoa Rajerison et Sébastien Boyer.

SOMMAIRE

Rappel des faits: Les Prisons de Madagascar sont à haut risque d'épidémie de peste. L'apparition d'une épidémie de peste dans les prisons peut provoquer un épisode important de peste urbaine par le déplacement d'humains, de rongeurs et de puces potentiellement infectés. Le contrôle des puces et des rongeurs est essentiel pour prévenir la peste, en diminuant les contacts humains avec les réservoirs et les vecteurs de la peste. Le traitement par insecticides est l'étape importante offerte pour contrôler les puces de rat qui transmettent la maladie des rongeurs infectés à l'Homme. La mise en œuvre d'une stratégie adaptée pour contrôler les puces devrait s'appuyer sur la condition de vulnérabilité aux insecticides de la population ciblée. Dans le cadre de la campagne de prévention de la peste dans les prisons, nous avons mené une recherche sur la résistance aux insecticides sur le *Xenopsylla cheopis,* la puce de rat.
Les méthodes: Des puces ont été recueillies sur des rats attrapés dans six prisons du Madagascar. Elles ont été exposées à des papiers filtres traités à l'insecticide et nous avons enregistré des mortalités, en suivant le protocole de l'Organisation mondiale de la Santé.
Les résultats : Les puces recueillies dans les prisons ont démontré différents profils de résistance, tandis qu'un haut niveau de résistance aux insecticides testés a été décrit dans la prison d'Antanimora, située au cœur d'Antananarivo, la capitale du Madagascar.
Les conclusions: Cette découverte est alarmante dans un contexte de santé publique, en sachant que l'efficacité du contrôle des puces pourrait être en péril à cause de la résistance aux insecticides. Afin d'établir un contrôle plus précis des puces de rat dans les prisons, les principales recommandations sont fondées sur la surveillance en continue de la vulnérabilité des puces aux insecticides, de la rotation des insecticides et du développement de nouvelles méthodes de contrôle des puces.

Translated from English version into French by Nathalie Thompson, through

Риск чумы в уязвимых общинах: оценка восприимчивости *блохи крысиной южной* к воздействию инсектицидов в малагасийских тюрьмах

Аделаид Миаринжара, Жан Верген, Жан Марсель Каваруганда, Миноарисоа Ражерисон, Себастьен Буайе

Аннотация

Предпосылка: Тюрьмы на Мадагаскаре подвергаются высокому риску вспышки чумы. Возникновение эпидемии чумы в тюрьмах может вызвать значительное появление чумы в городе из-за передвижения потенциально зараженных людей, грызунов и блох. Контролирование грызунов и блох имеет основное значение в предотвращении чумы, уменьшая контакт человека с естественными резервуарами чумы и ее переносчиками . Обработка инсектицидами является ключевым шагом, доступным для контроля крысиных блохи, которые передают болезнь от инфицированных грызунов человеку. Внедрение стратегии, адаптированной к контролю блох, должно опираться на уровень восприимчивости целевого населения к инсектицидам. В целях кампании предотвращения чумы в тюрьмах мы провели исследование резистенции *блохи крысиной южной* к инсектицидам.

Методы: Блохи были собраны на крысах, пойманых в шести тюрьмах Мадагаскара. Они были подвержены действию фильтровальной бумаги, обработанной инсектицидом, и смертность была зарегистрирована согласно протоколу Всемирной организации здравоохранения.

Результаты: Собранные в тюрьмах блохи имели различные характеристики резистентности, в то время как протестирован высокий уровень резистентности к инсектицидам, описаный в Антанимурской тюрьме, расположеной в самом центре Антананариву, столице Мадагаскара.

Выводы: Обнаружение этого факта вызывает тревогу в контексте общественного здравоохранения, зная, что резистентность к инсектицидам может поставить под угрозу эффективность контроля над блохами. Чтобы создать более точный контроль над крысиными блохами в тюрьмах, главные рекомендации базируются на непрерывном мониторинге восприимчивости блох к инсектицидам, ротации инсектицидов и разработке нового метода контроля над блохами.

Translated from English version into Russian by Dariia Moss, through

Riesgo de plaga en una comunidad vulnerable: evaluación de la suceptibilidad de la *Xenopsylla cheopis* a insecticidas en las cárceles de Madagascar

Adélaïde Miarinjara, Jean Vergain, Jean Marcel Sébastien Boyer Kavaruganda, Minoarisoa Rajerison,

RESUMEN

Antecedentes: Las cárceles en Madagascar tienen un riesgo alto de que se genere una peste. Una peste epidémica en las cárceles puede causar un episodio significativo de peste urbana a través del movimiento de personas, pulgas y roedores potencialmente infectados. Los controles de roedores y pulgas son esenciales en la prevención de la peste, al reducir el contacto humano con vectores y reservorios de la peste. El tratamiento insecticida es el paso clave disponible para el control de las pulgas en ratas que transmiten la enfermedad de los roedores infectados a las personas. La implementación de una estrategia adaptada de control de pulgas debe basarse en el estado de susceptibilidad al insecticida de la población objetivo. Para hacer una campaña de prevención de peste en las prisiones, realizamos un estudio de resistencia al insecticida en la *Xenopsylla cheopis,* la pulga de la rata.

Métodos: Se obtuvieron ratas atrapadas en seis cárceles de Madagascar. Se las expuso a papeles filtro tratado con insecticida y se registró la mortalidad según el protocolo de la Organización Mundial de la Salud.

Resultados: Las pulgas recogidas en las cárceles tenían patrones de resistencia diferentes, mientras que se describió un nivel alto de resistencia a los insecticidas probados en la cárcel de Antanimora, ubicada en el centro de Antananarivo, la capital de Madagascar.

Conclusiones: Este hallazgo es alarmante en el contexto de la salud pública, al saber que la resistencia a los insecticidas comprometería la efectividad del control de pulgas. Para establecer un control más preciso de las pulgas de rata en las cárceles, las principales recomendaciones se basan en monitorear en forma continua la sensibilidad de las pulgas al insecticida, cambiar de insecticida y desarrollar un método nuevo para el control de pulgas.

Translated from English version into Spanish by Lidia Lariño, through

TRANSLATORS
WITHOUT BORDERS
